# Recyclable Catalysts for Alkyne Functionalization

**DOI:** 10.3390/molecules26123525

**Published:** 2021-06-09

**Authors:** Leslie Trigoura, Yalan Xing, Bhanu P. S. Chauhan

**Affiliations:** 1 Department of Chemistry, William Paterson University of New Jersey, 300 Pompton Road, Wayne, NJ 07470, USA; trigoural@wpunj.edu; 2 Engineered Nanomaterials Laboratory, Department of Chemistry, William Paterson University of New Jersey, 300 Pompton Road, Wayne, NJ 07470, USA

**Keywords:** green catalysts, nanosystems, nanoscale catalysts, catalyst modulation, alkyne functionalization, coupling reactions

## Abstract

In this review, we present an assessment of recent advances in alkyne functionalization reactions, classified according to different classes of recyclable catalysts. In this work, we have incorporated and reviewed the activity and selectivity of recyclable catalytic systems such as polysiloxane-encapsulated novel metal nanoparticle-based catalysts, silica–copper-supported nanocatalysts, graphitic carbon-supported nanocatalysts, metal organic framework (MOF) catalysts, porous organic framework (POP) catalysts, bio-material-supported catalysts, and metal/solvent free recyclable catalysts. In addition, several alkyne functionalization reactions have been elucidated to demonstrate the success and efficiency of recyclable catalysts. In addition, this review also provides the fundamental knowledge required for utilization of green catalysts, which can combine the advantageous features of both homogeneous (catalyst modulation) and heterogeneous (catalyst recycling) catalysis.

## 1. Introduction

Alkyne functionalization methods constitute one of the most relevant topics in organic synthesis and has resulted in numerous advancements over several years. Alkynes are readily available building blocks for organic synthesis because carbon–carbon multiple bonds can be functionalized to a myriad of structures. Direct functionalization of alkynes is a powerful approach for the construction of various complex organic structures because of its high efficiency in the cascade formation of carbon–carbon and carbon–heteroatom bonds [1,2,3,4,5].

There is a key interest in developing new systems and conditions to improve the efficiency for these reactions. Apart from the tremendous attention that this subject in chemistry has achieved, there are still certain limitations that prevent its advanced adoption for applications in industry. Some of these drawbacks include cost, efficiency, use of toxic metals, tedious catalyst recoverability, metal leaching, low regioselectivity, poor functional group tolerance, the need for excess amounts of reagents, and low catalytic activity and stability. 

**Figure 1:**
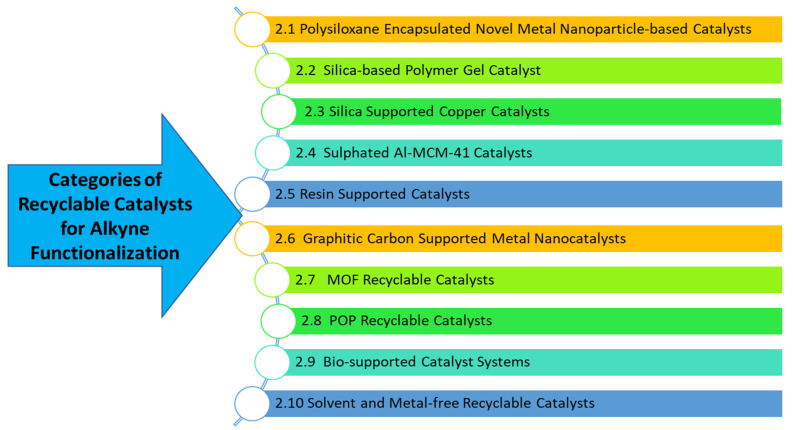
Categories of recyclable catalysts in alkyne functionalization highlighted in this review.

The recyclability of the catalysts in organic synthesis is important to study, especially when looking to the applicability of reactions in industrial settings. Herein, we have provided a comprehensive survey and analysis of the recent and developmental work in alkyne functionalization using a variety of different classes of recyclable catalysts. Each of the reported catalysts has been reported to result in transformations in high yields and to have the ability to be recovered and recycled from the reaction, up to or more than four times. The catalytic activity of each catalytic system reported seems to maintain its stability, even after reuse, and has been shown to perform competitively when compared to traditional methods used to carry out the same proposed reactions. In this review, we have highlighted polysiloxane-encapsulated novel metal nanoparticle-based catalysts, silica–copper-supported nanocatalysts, graphitic carbon-supported nanocatalysts, metal organic framework (MOF) catalysts, porous organic framework (POP) catalysts, biomaterial-supported catalysts, and metal-/solvent-free recyclable catalysts (Figure 1).

**Figure 2:**
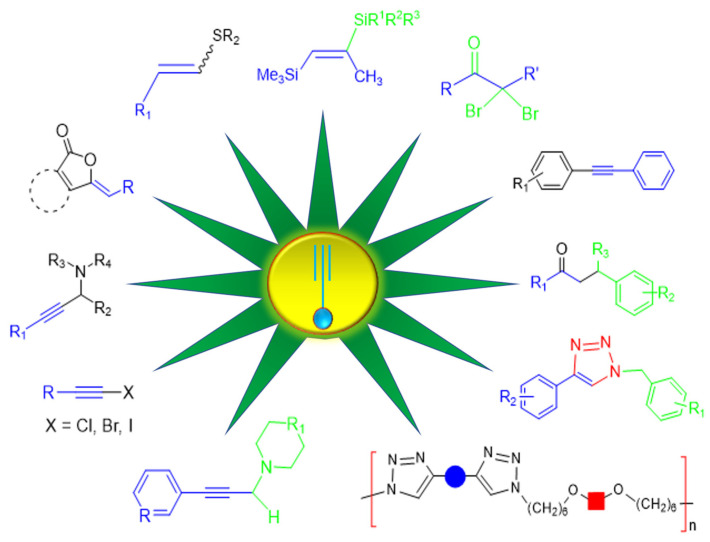
The wide array of alkyne functionalized products achieved through the reactions focused on in this review via recyclable catalysts.

Additionally, amongst the highlighted catalysts, a variety of alkyne functionalization reactions have been studied to yield several functionalized products, such as symmetrical and unsymmetrical 1,3-diyne motifs, vinyl silanes, dihalogenated ketones, cross-coupled functionalized alkynes, 1,4-disubstituted-1,2,3-triazoles, polytriazoles, propargylamines, 1,2,3-triazoles, 1-haloalkynes, alkylidenefuranones, thioesters, and others (Figure 2).

The successful replacement of single-use catalysts with recyclable catalysts has been demonstrated throughout all the mentioned work, allowing the reactions to have a broad substrate scope, good regioselectivity, and excellent yields, as would any traditionally used catalyst, while simultaneously providing the advantages of simple removal and reusability. Although previously published reviews may highlight the use of recyclable catalysts, the purpose of this review will be to showcase these new and developing catalytic approaches in the field of alkyne functionalization.

## 2. Catalyst Potential and Recyclability

### 2.1. Polysiloxane Encapsulated Novel Metal Nanoparticle-Based Catalysts

**Figure 3:**
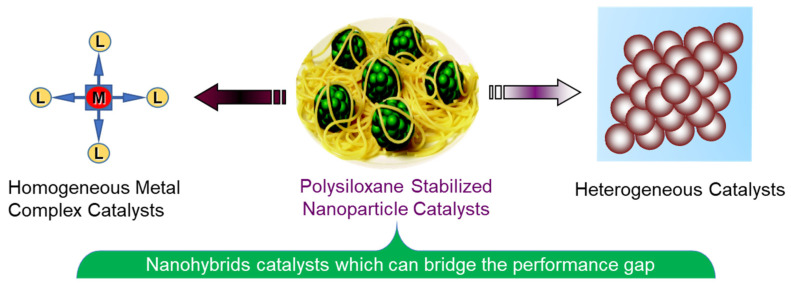
Polysiloxane-stabilized nanoparticle catalysts with both homogenous and heterogenous catalytic characteristics.

Creation of recyclable catalysts permits one to help develop green chemical processes which at a larger scale positively impact our environment as well as contribute to the development of a new economy. Chauhan et al. have been developing new green catalysts which have shown unparalleled efficiency and selectivity in various transformations such as hydrosilylation, polymer modification, oxidative coupling reactions and alcoholysis processes of hydrosilanes, to name a few. The basic framework in these new catalysts pivots on the fact that support materials are created in such a fashion that these catalysts behave as heterogenous catalysts in terms of recyclability but at the same time provide a very high degree of selectivity as homogeneous catalysts (Figure 3). 

The physical encapsulation of catalysts on silica-based materials is very well-known but suffers from drawbacks such as leaching, which leads to a decrease in catalyst efficiency and selectivity.

The major advantageous features of such catalysts are:

1. **Nanoclusters possess very high surface area**

The relative high surface-to-volume ratio provides a larger surface for substrates to interact with nanoparticle catalysts, which makes them as active as corresponding molecular complexes as catalysts.

2. **Nanocluster surfaces can be tailored**

The recent developments in surfactant tailoring have opened up new opportunities for activity and selectivity tuning as opposed to heterogeneous catalysts.

3. **Nanoclusters precipitate out of the reaction mixture after catalysis**

Proper tailoring of the polysiloxane as well as other nanocatalyst stabilizers permit recycling of the catalysts by simple centrifugation, which makes them very attractive as recyclable catalysts.

4. **Catalytic activity of nanoclusters is not affected after repeated cycles**

Since the leaching of the nanoparticle catalysts can be avoided by proper tailoring of the surfactant stabilizers, it is observed that the nanoparticle catalysts remain active and selective, even after various cycles of catalysis.

**Scheme 1:**
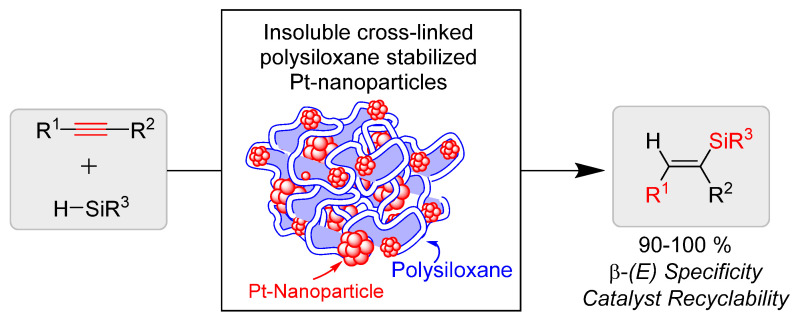
Hydrosilylation reaction of functional alkynes for the synthesis of various vinyl silane products (adopted from [6]).

In the industrial-scale production of organic/inorganic hybrid functional materials, vinylsilanes and their derivatives are considered to be key building blocks. The green routes to the production of such building blocks have been explored since a very long time. In 2017, Chauhan’s group disclosed the production and utilization of a mild, predictable, facile and widely applicable hybrid-phase platinum nanoparticle-based green catalysts to produce industrially relevant vinyl silane building blocks [6] (Scheme 1).

**Scheme 2:**
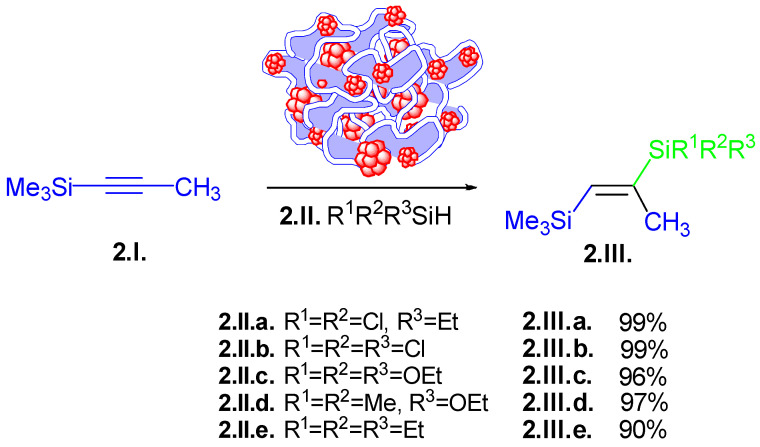
Selective hydrosilylation of mono- and di-substituted alkynes to yield vinylsilanes via a polysiloxane-stabilized platinum nanoparticle recyclable catalyst (adopted from [6]).

This is a mild and efficient approach for the selective hydrosilylation of mono- and di-substituted alkynes to synthesize vinyl silanes through the use of a polysiloxane-stabilized platinum nanoparticle recyclable catalyst (Scheme 2). This reaction and catalyst system takes advantage of its heterogenous properties for its effectiveness as a recyclable and reusable catalyst. The scope of this hydrosilylation reaction includes a variety of acetylenes showcasing the wide tolerability of its catalytic capabilities (**2.I.**).

One of the major highlights of this reaction and catalyst is its regioselectivity, which causes it to favor the β-E isomer product formation, as observed in their studies (**2.II.a–e**). Exploration of their substrate scope involves the incorporation of silanes with electron-donating and -withdrawing groups, acetylenes with electron-donating and -withdrawing groups, and disubstituted acetylenes, all of which resulted in good to excellent yields (**2.III.a–e**). The catalyst is reported to have a facile recovery and can be reused up to five consecutive times with no noticeable decrease in catalytic activity. Based on the findings, it is notable to point out that this catalyst provides high selectivity, mild reaction conditions, diverse functional group compatibility, and excellent recyclability.

### 2.2. Silica Based Polymer Gel Catalysts

**Scheme 3:**
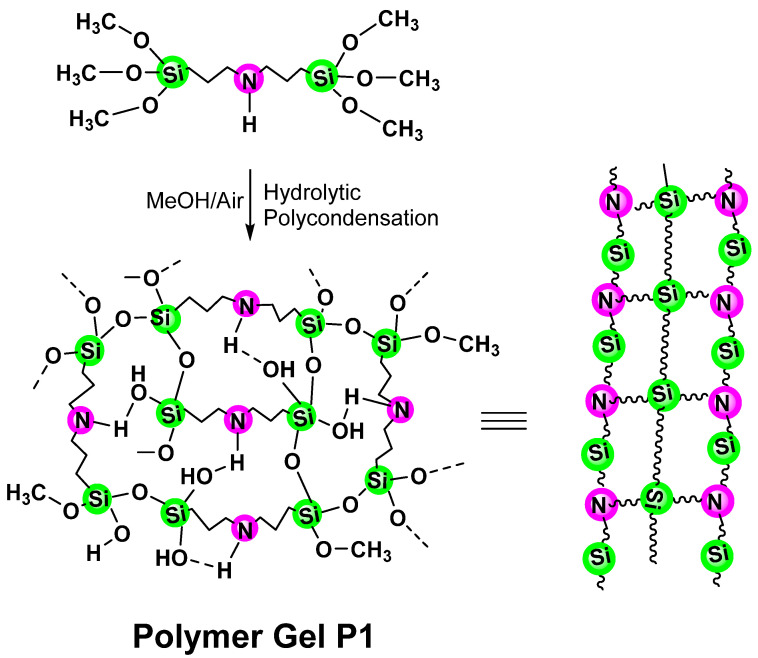
Synthesis and structure of polymer gel P1 (derived from [7]).

In 2018, Chauhan et al. [7] synthesized an amino-bridged silsesquioxanes sol gel polymer (**Polymer Gel P1**) by cross-linking amine containing monomeric silanes. In this case, their goal was to create methoxy-containing gels with complexing agents such as amines. In this publication, they describe that the hydrolytic polycondensation of the precursor bis [3-(trimethoxysilyl)propyl]amine in wet methanol for six hours under ambient reaction conditions and in open air produces colorless silica gel, P1, which still has a significant amount of Si-alkoxy groups (Scheme 3).

**Scheme 4:**
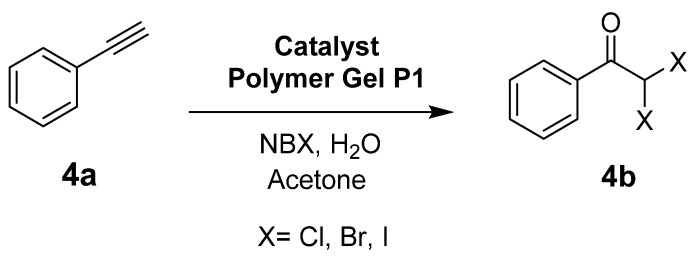
One-pot alkyne halo-functionalization to synthesize α,α-dihalogenated ketones through use of the recyclable catalyst polymer gel P1 (adopted from [7]).

Chauhan et al. [7], in collaboration with Xing’s group, discovered that polymer gel P1 indeed functions as an efficient recyclable catalyst for the one-pot halo-functionalization of alkynes to produce α,α-dihalogenated ketones obtained with both a good yield and excellent selectivity over any other by products when acetone was used as the solvent (Scheme 4).

This difunctionalization reaction to yield various di-halogenated ketones involves the use of a heterogenous catalyst, allowing it to catalyze the reaction, be removed from the reaction mixture, and to be reused, which makes it a sustainable alternative to perform these reactions.

**Scheme 5:**
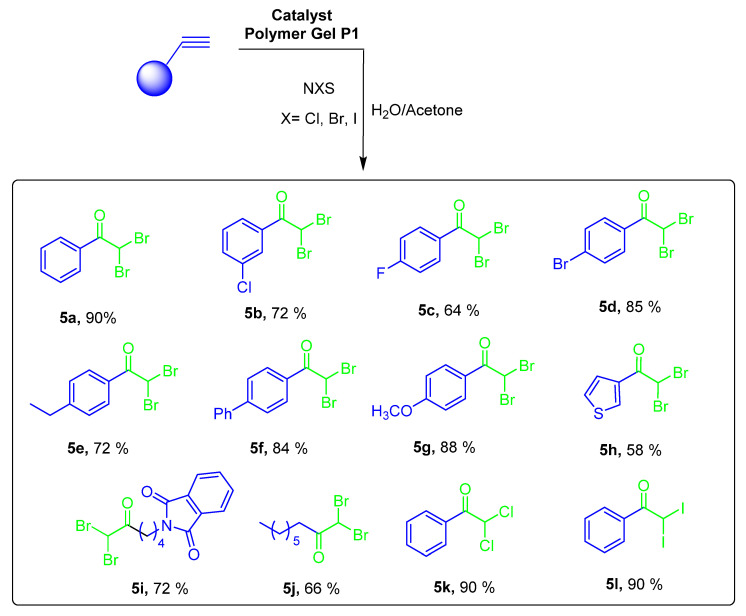
Substrate scope for the synthesis of a variety of α,α-dihalogenated ketones through use of this one-pot alkyne halo-functionalization via catalyst polymer gel P1 (derived from [7]).

Based on this study, the dibromination (Scheme 5a–i), dichlorination (Scheme 5k), and diiodination (Scheme 5l) of alkynes can be performed on a variety of substrates with high selectivity and excellent functional group compatibility (Scheme 5). 

Aromatic substrates with electron-withdrawing and electron-donating groups on various positions of the ring were tested and resulted in excellent yield (Scheme 5a–i,k–l). They also explored the reaction’s tolerance with alkyl substrates, which also resulted in good yields (Scheme 5i–j). Additionally, internal alkynes were well-tolerated, resulting in clean transformations and less reactive aliphatic compounds that were also decently compatible. They also explored the recyclability of the sol gel polymer catalyst and the studies revealed that it could be used 10 times before any significant decrease in activity.

### 2.3. Silica Supported Copper Catalysts

The study of different support systems immobilized with copper ions is important for the development of innovative, sustainable, and recyclable catalysts. Copper catalysts play a crucial role in many organic chemistry reactions, and when immobilized onto these support materials, they create heterogeneous catalysts with much more capabilities and catalytic activity. Aside from an increase in efficiency, the advantage of incorporating heterogenous catalysis is the ability to recover and remove the catalyst from the reaction mixture after completion, one of the remaining major challenges of current homogenous catalysts. In taking advantage of this property, the recyclable property allows one to reuse the catalyst efficiently for a certain number of cycles without worrying about a substantial decrease in catalytic activity. For example, previously in 2013, Jacob et al. [8] reported the use of porous glasses as novel support systems for the immobilization of copper salts to catalyze a microwave-assisted azide-alkyne cycloaddition click reaction. Through their heterogenous recyclable catalyst, they were able to carry out these reactions efficiently, in water and in short reaction times, and demonstrated their catalyst to be capable of recovery and reuse up to four cycles with no significant loss of activity, which was a leading development when compared to other similar studies.

**Scheme 6:**
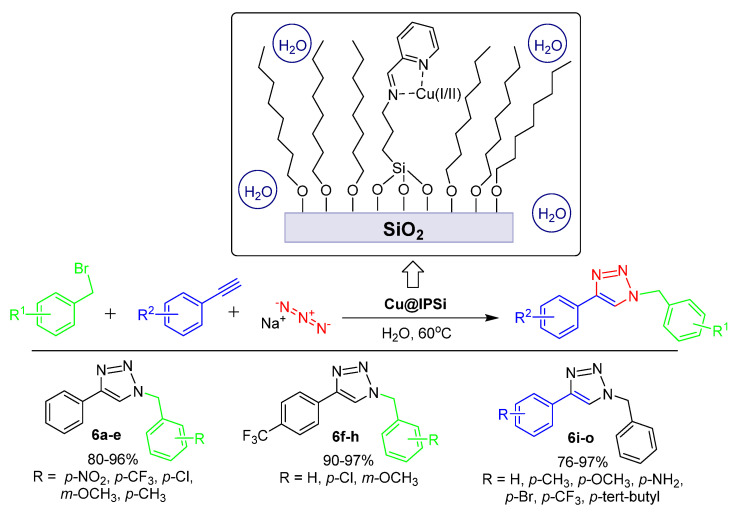
Copper-catalyzed one-pot azide-alkyne cycloaddition reaction through heterogeneous Cu-reverse phase silica gel catalyst (derived from [9]).

Additionally, in 2018, Lim et al. [9] conducted a copper-catalyzed one-pot azide-alkyne cycloaddition reaction using two heterogenous Cu-reverse phase silica gel catalytic systems (Scheme 6).

The catalysts were composed of reverse-phase silica gels which contained hydrophobic end caps in the alkyl group chains. Since this one-pot click azide-alkyne cycloaddition reaction takes place in water as the choice of solvent, the hydrophobic properties of the end caps will push and move the reactants continuously towards the active catalytic site, where the reaction can efficiently take place, until all of the reactants are transformed, or the reaction is stopped. The researchers synthesized both Cu(I) and Cu(II) catalysts, both of which proved to be efficient in the reaction transformations; however, Cu(II)@pNIPAM-VP was reported to be slightly more reactive than Cu(I)@pNIPAM-VP, when compared. 

To test the tolerance of these catalysts, each was used to catalyze a mixture of substrates consisting of aromatic substituents, including electron-withdrawing and electron-donating groups. Various positions of the benzyl bromide substrate were tested with phenylacetylene yielding between 80 and 96% (Scheme 6a–e). Additionally, other benzyl bromide derivatives were tested with a *p*-trifluoromethyl-phenylacetylene resulting in excellent yields between 90 and 97% (Scheme 6f–h). A variety of terminal alkyne substrates were tested with benzyl bromide as the constant model substrate and also resulted in excellent yields between 76 and 97% (Scheme 6i–o). The catalysts are reported to be recyclable from the reaction and easily removed through hot filtration, and can be reused several times with very little effect to yield. To test for leaching, Lim et al. used ICP tests and confirmed that Cu(I)@pNIPAM-VP exhibited no copper leaching; however, Cu(II)@pNPAM-VP did display copper leaching after the third recycled use, leading to a decrease in yield after the third reuse. Taking advantage of these catalyst’s recyclability and hydrophobic properties, allowing this organic reaction to be successfully performed in water, makes it a green and sustainable alternative method.

**Scheme 7:**
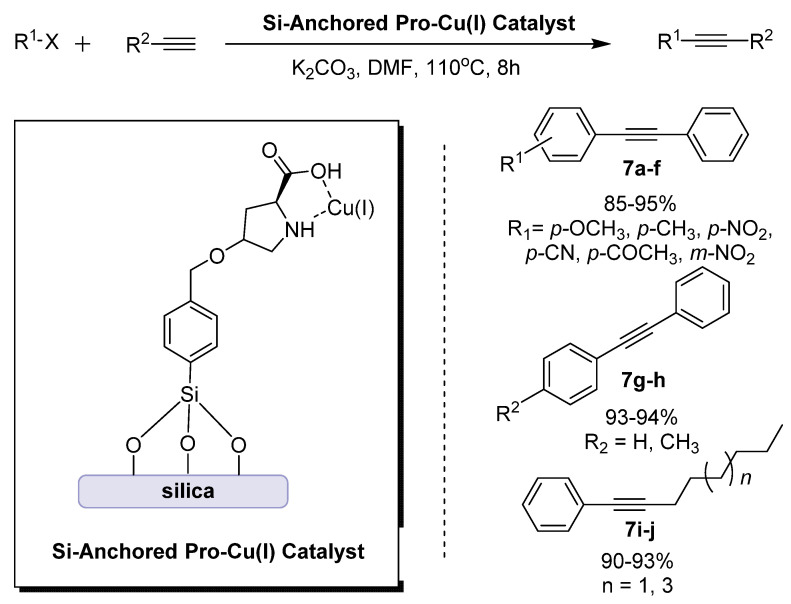
Sonogashira reaction through the use of silica-anchored proline-copper (I) hybrid recyclable catalyst (adopted from [10]).

Previously in 2008, Wang and partners [10] reported on the use of copper (I) with proline anchored onto silica as a sustainable hybrid material that could act as a recyclable catalyst to carry out the Sonogashira reaction (Scheme 7).

This cross-coupling reaction was performed on aryl halides, specifically iodine- and bromine-containing substrates, along with terminal alkynes to yield the cross-coupled alkyne-functionalized products efficiently. The Sonogashira reaction is known for its successfulness; however, one of its major drawbacks is its current and most common use of palladium. This not only poses a potential issue with regards to its toxicity, in the aspect of green catalysis, but one may additionally take into consideration the high expense for this class of catalysts. For these reasons, greener and more affordable alternative methods to carry out this reaction are of great interest. Several functionalities were tolerated through these reaction conditions, resulting in excellent yields. Both electron-poor and electron-rich aryl iodides and bromides reacted well under the conditions and were successfully converted to product with excellent yields above 90% (Scheme 7a–e). Out of all the aryl halides tested, the *m*-nitro-substrate resulted in the lowest yield at 85% (Scheme 7f), when compared to the others, potentially resulting from steric hindrance. A few terminal alkyne substrates were also tested, out of which the aromatic substrates resulting in the successful formation of product between 93 and 94% (Scheme 7g–h). Aliphatic terminal alkynes were also reacted to test the limits of the reaction scope and were found to also yield positive results of 90 to 93% (Scheme 7i–j). Wang et al. reported that the silica-anchored proline-copper(I) catalyst could be recovered after each reaction through simple vacuum filtration, with no need of further purification, and after a series of tests, the recyclable catalyst was shown to be reusable for up to six reactions before any significant decrease in catalytic activity could be observed.

**Scheme 8:**
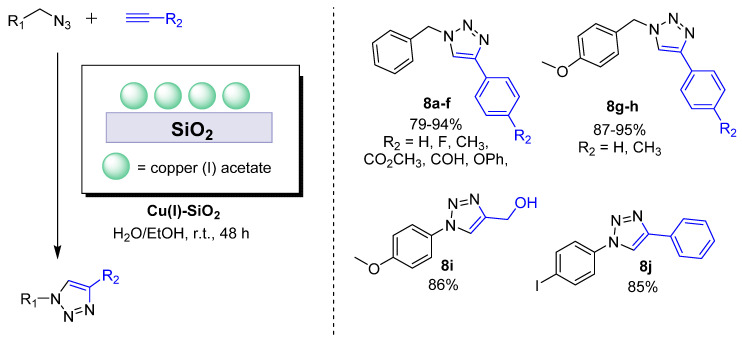
Synthesis of 1,4-disubstituted-1,2,3-triazoles via copper (I) acetate-anchored silica-catalyzed azide-alkyne cycloaddition reactions (derived from [11]).

Recently in 2019, Aflak et al. [11] have reported on their use of a heterogenous recyclable catalyst consisting of copper(I) acetate placed on silica to catalyze azide-alkyne cycloaddition reactions for the synthesis of 1,4-disubstituted-1,2,3-triazoles (Scheme 8).

The heterogenous aspect of this catalytic system plays a critical role in its ability to act as a reusable, recyclable catalyst. If this were not the case, the system would have thermodynamic instability and likely oxidation of the copper metal, requiring additional stabilizing ligands, and not meeting the green aspects of the transformation methodology. The catalyst was synthesized by using an impregnation technique and dissolving copper (I) acetate in acetone, adding the silica, and stirring the mixture overnight under room temperature conditions. Following that, the mixture is filtered, and oven dried overnight. According to the optimal conditions, this reaction is best suited in water/ethanol and at room temperature, which is ideal in terms of sustainability. 

To showcase the competence of the reaction, Aflak et al. tested a substrate scope for the reaction including various organic azides and different alkyne substrates. They ran click reactions of aromatic azides with para-substituents with 87 to 95% yields (Scheme 8g–h) and different aromatic terminal alkynes with para-substituents, resulting in good yields between 79 and 94% (Scheme 8a–f,j). An aliphatic terminal alkyne containing an alcohol group was also tested to push the boundaries of the scope and resulted in a yield of 86% (Scheme 8i). The synthesized immobilized catalyst was shown to be simply removed after the reaction was completed through filtration and solvent washing with a small amount of diethyl ether. The researchers demonstrated through their analysis that the heterogenous catalyst could be recycled up to five consecutive times before a notable decrease in yield due to copper leaching after the fifth reaction use. 

The coupling of terminal alkynes to yield these symmetrical and unsymmetrical 1,3-diyne motifs, commonly found in natural products and other bioactive compounds, was discovered in 1869 by Glaser [12]. Based on traditional methods, this coupling requires the use of palladium and oxidative agent additives [13], which poses potential environmental and expense concerns. There have been many recent improvements to find new sustainable and greener methodologies that can efficiently carry out these reactions using transition metal catalysis, such as the work reported by Holganza et al. [14] in 2019, where they used a Cu(II) catalyst with air as the oxidizing agent under room temperature. However, works such as this present new effective alternative are slightly limited in the sense that they are efficient but only single reactions and do not involve recyclable catalysts that can be used for multiple times. These single reactions may be useful for synthetic transformations, but developments of materials that can act as recyclable catalysts with efficient catalytic activity to carry out these transformations is also needed for its environmentally friendly approach and possible applications in industry. 

**Scheme 9:**
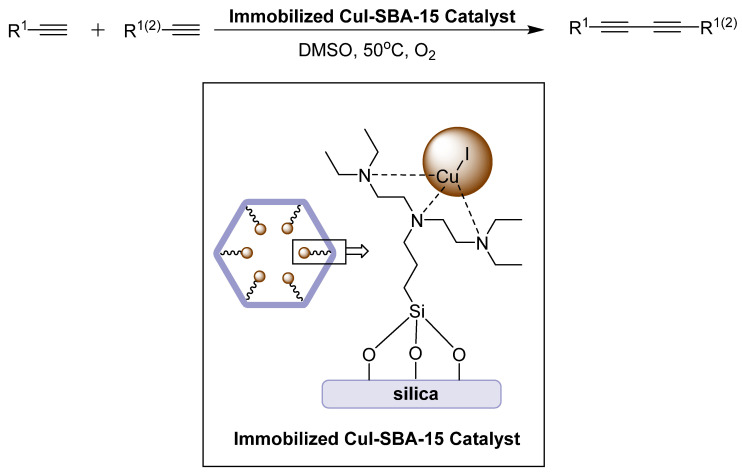
Homocoupling and heterocoupling of terminal alkynes via immobilized CuI-SBA-15 catalyst for synthesis of symmetrical and unsymmetrical 1,3-diynes (adopted from [15]).

Ma e tal. [15] previously reported, in 2012, the use of immobilized CuI SBA-15 as a catalyst for the efficient homocoupling and heterocoupling of terminal alkyne and without the need for base, for the successful synthesis of both symmetrical and unsymmetrical 1,3-diynes (Scheme 9).

Optimal conditions for this reaction were discovered to require the catalyst in the presence of DMSO, oxygen as an oxidizing agent, and to be conducted at 50 °C. Once the optimal conditions were obtained, the researchers used this condition to study the scope of their reaction and the catalytic efficiency of their catalyst using a series of terminal alkynes. They started by conducting several homocoupling reactions to yield symmetrical 1,3-diyne products, including substrates with electron-rich and electron-poor groups, as well as both electron-donating and -withdrawing groups on those aromatic compounds. These aromatic homocoupling products resulted in yields between 82 and 99%. Aliphatic substrates were also tested, but resulted in lower yields of 46 to 82% as determined through GC-MS. For the synthesis of unsymmetrical 1,3-diynes through heterocoupling reactions, the substrates tested consisted of aromatic compounds with different electron-donating and -withdrawing substituents and provided yields between 45 and 56%, with little by-product formation. Recyclability tests for this catalyst demonstrated its simple recovery after reaction completion, through simple filtration, and its ability to be reused up to five times before seeing any weighty decrease in catalytic activity, due to significantly more copper leaching after the fifth use.

### 2.4. Sulphated Al-MCM-41 Catalysts

**Scheme 10:**
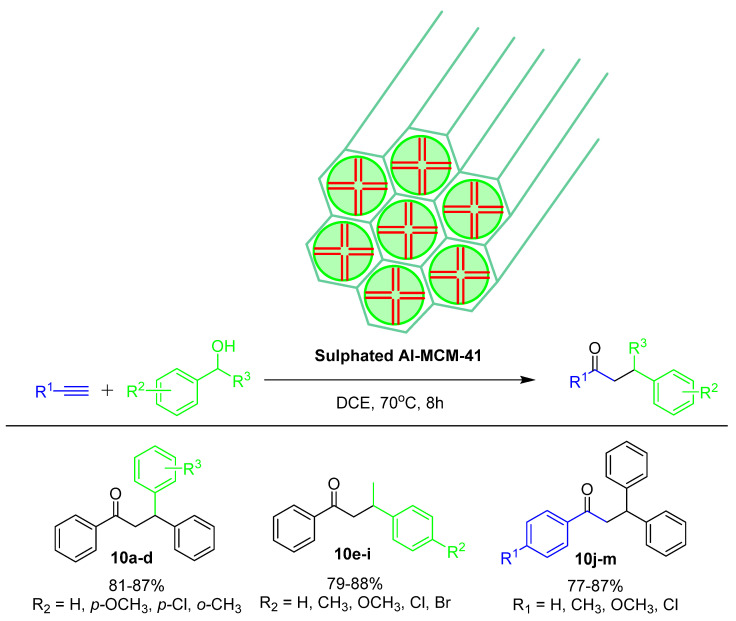
Alkyne alcohol addition catalyzed by sulphated Al-MCM-41 (adopted from [16]).

In 2018, Wagh et al. [16] utilized a sol-gel and impregnation method to synthesize sulphated Al-MCM-41 in order to study its applications as a recyclable catalyst for efficient alcohol addition to alkynes (Scheme 10).

The catalyst is composed of aluminum and silica components which were supplied to the material through tetraethyl orthosilicate and aluminum isopropoxide. During the synthesis, Lewis acid sites are formed due to a deficit in partial charge caused by the addition of Al^3+^ to the originally stable MCM-41 silica framework, creating Al-MCM-41. During impregnation, the sulphate is infused in the Al-MCM-41, where it coordinates itself within the framework. As a result, strong Bronsted acid sites are formed in the sulphated Al-MCM-41 catalysts, which is directly correlated to its efficiency for direct alcohol addition to alkynes.

In order to test the functional group tolerance of the reaction using this Al-MCM-41 catalyst, the substrate scope included a range of different secondary alcohols which were ran with phenylacetylene as a model substrate. Among these secondary alcohols tested, the scope included aromatic groups with different substituents in the ortho and para positions, providing good yields of 81 to 87% (Scheme 10a–d). Aliphatic alcohol groups were also tested and yielded similar successful results when combined with different adjacent aromatic groups, yielding between 79 and 88% (Scheme 10e–i). Furthermore, a few terminal alkynes were tested in the reaction, mostly consisting of aromatic phenylacetylene derivatives containing electron-withdrawing and -donating groups at the para position. These alkyne substrates had positive results in the addition of the alcohols, providing yields of 77 to 87% (Scheme 10j–m). Along with its catalytic activity in the direct addition of alcohols, the authors additionally demonstrated the versatility and efficiency of sulphated Al-MCM-41 as a recyclable catalyst for the coupling of alcohols to styrene derivatives. After studies on reusability of the catalyst, it was found that the yield decreases slightly after the sixth recycled use, resulting from its decline in acidic potency. Due to its simplicity, selectivity, mild approach, and recyclability, this catalyst displays great potential for this and many future transformations.

### 2.5. Resin Supported Catalysts

**Scheme 11:**
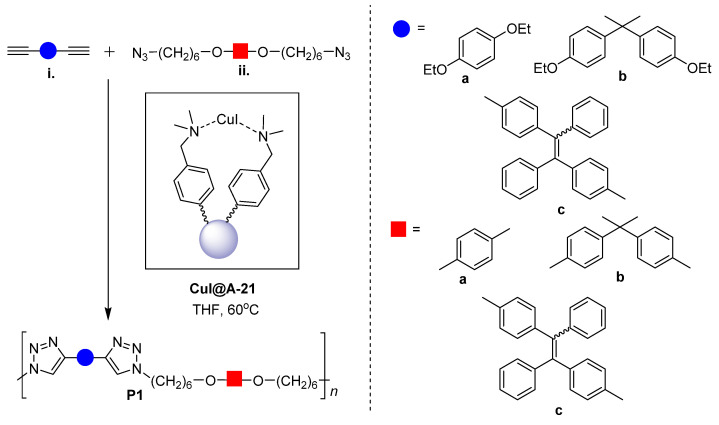
CuI@A-21 supported catalysis of alkyne-azide click polymerization for the production of polytriazoles (derived from [17]).

Previously, in 2014, Wu et al. [17] reported on the use of CuI@A-21 (Amberlyst^®^ A-21 resin)-supported catalyst for the efficient catalysis of alkyne-azide click polymerization for the synthesis of polytriazoles (Scheme 11).

The benefit of using CuI@A-21 as a recyclable catalyst to perform this polymerization reaction is that there will not be a coordination of leftover copper residues with the created triazole structures present, which would be the case with alternative Cu(I) catalysts, making the catalyst removal difficult and therefore recyclability almost impossible. By avoiding this, the CuI@A-21 is capable of being removed after completion of the reaction and able to be reused in another reaction. Synthesis of this catalyst is simple; it involves creating a mixture of CuI and the A-21 resin with acetonitrile as a solvent. Allowing this to stir at room temperature under nitrogen gas for 17 h will yield the desired CuI@A-21 catalyst with about a 94% yield. A variety of different substrate units were used within the monomers for the polymerization of several diynes and diazides. These substrate units consisted of various aromatic groups and of varying molecular weights, with the highest being 69,600 and a reported polydispersity of < 3.25. Using the CuI@A-21 catalyst in the presence of THF at 60 °C, the product polytriazoles (P1) were achieved in good yields between 65 and 98%. Recyclability studies by the researchers demonstrated that the catalyst could be reused at least four times before seeing a significant loss in yield and catalytic activity. They attribute this advantage to the catalyst’s ability to chelate copper and nitrogen that is present on the surface of the resin-supported catalyst, allowing there to be a control or retardance for the leaching of copper throughout each use.

**Scheme 12:**
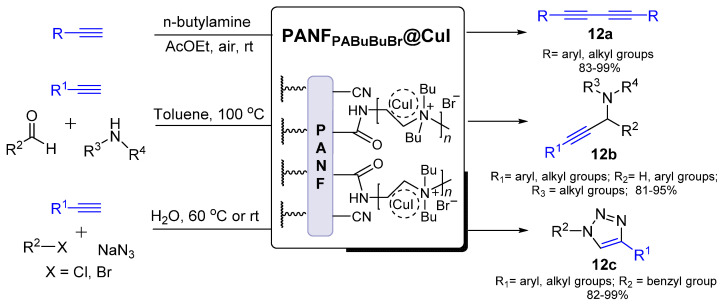
PANF_PABuBuX_@CuX (where X = I and Br) as a recyclable catalyst for (**a**) terminal alkyne homocoupling; (**b**) alkyne, aldehyde, and amine cycloaddition (A3 coupling) reactions; and (**c**) alkyne-azide cycloaddition click chemistry (CuAAC) reactions (derived from [18]).

The use of copper-supported catalysts has gained attention and favor due to its affordable cost, efficient catalytic activity, wide tolerability for various organic transformations and functionalities, and recent advances in the support systems used. In 2018, Hu et al. [18] synthesized novel fiber-supported polyquaterniums@Cu(I) (PANF_PABuBuX_@CuX, where X = I and Br), to be used as effective recyclable catalysts for efficient (i) terminal alkyne homocoupling; (ii) alkyne, aldehyde, and amine cycloaddition (A3 coupling) reactions; and (iii) alkyne-azide cycloaddition click chemistry (CuAAC) reactions (Scheme 12).

This fiber-supported polyquaterniums@Cu(I) catalyst was synthesized via a five-step synthetic process involving: amination using polyethylene polyamine, salinization with the introduction of PANF_PA_ and 1-bromobutane, neutralization with sodium carbonate solution, quaternization to provide the PANF-supported polyquaterniums, and finally chelation with CuX (either CuI or CuBr) to yield the desired catalyst. 

First, the homocoupling of terminal alkynes was catalyzed using this synthesized recyclable catalyst in the presence of n-butylamine and ethyl acetate, under room temperature and air for the synthesis of 1,3-diynes. Various aromatic, aliphatic, and cyclic, terminal alkyne substrates were tested, which resulted in yields between 83 and 99% (Scheme 12a). Secondly, the catalyst was used in the presence of toluene at 100 °C to perform a series of A^3^ coupling reactions to produce propargylamines. In this case, three varying substrates were tested: different alkynes, aldehydes, and amine substrates. For each test, all variables would remain constant, and one substrate would be tested at a time. The model substrates that remained constant for each of the three categories of substrates were phenylacetylene, benzaldehyde, and pyrrolidine. For the alkynes, an aromatic, aliphatic, and para-substituted substrate were tested, resulting in 81 to 94% yields (Scheme 12b). When the aldehydes were tested, aromatic and aliphatic substrates were tested, along with groups with electron-withdrawing and -donating substituents, yielding between 92 and 94% (Scheme 12b). Finally, the synthesis of various 1,2,3-triazoles via click chemistry CuAAC reactions in water were catalyzed using this fiber-supported copper complex catalyst. Several terminal alkynes were tested, including phenylacetylene, aromatic substrates with electron-withdrawing and -donating groups, aliphatic chain, and ester containing substrates. Each of these was tested with bromo- and chloro-methylbenzene, resulting in yields between 85 and 99% when reacted at 60 °C for 15 min and between 82 and 98% when reacted at room temperature for 6 h (Scheme 12c). 

The flexibility, durability, and mechanical strength of this fiber provides it with an advantage for its future applications in industry and as a recyclable catalyst. During the recyclability studies, after the completion of each reaction, the researchers reported to wash the reaction vessels and directly proceed to use them for the following reaction without the need of additional treatment on the catalyst. The study reports that the synthesized catalyst was able to be reused 10 times without any noticeable decrease in catalytic activity (<5% yield loss between the 1st and 10th cycled use). It was also reported that the catalyst can be stored on shelves and still maintain its catalytic potential stable for up to four months before degradation and without need of complicated storage. Additionally, to complement its potential for use in industry, Hu et al. also performed a gram-scale test and found that the catalyst worked as efficiently with no adjustments necessary, yielding similar results, and no decrease in catalytic activity.

### 2.6. Graphitic Carbon Supported Metal Nanocatalysts

**Scheme 13:**
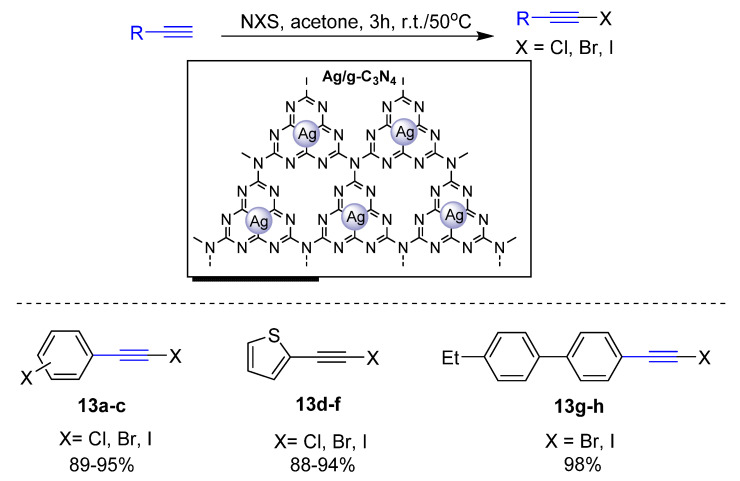
Halogenation of terminal alkynes to synthesize 1-haloalkynes via nano-Ag/graphitic carbon nitride (g-C_3_N_4_) recyclable catalyst (adapted from [20]).

The development of sustainable and efficient methods for the direct halogenation of terminal alkynes to synthesize and access 1-haloalkynes is essential, as they are relevant building blocks in organic synthesis [19]. Shi et al. [20] reported, in 2017, on the synthesis and use of nano-Ag/graphitic carbon nitride (g-C_3_N_4_) as a highly efficient recyclable catalyst system for the chlorination, bromination, and iodination of terminal alkynes to access these 1-haloalkynes (Scheme 13).

They synthesized this catalyst system by immobilizing Ag particles onto the surface of g-C_3_N_4_, via a one-step polymerization method using NaBH_4_ as a reducing agent. Optimal conditions for all three halogenation reactions were similar, aside from the temperature. It was discovered that bromination and iodination occurred efficiently at room temperature; however, chlorination yielded the best results when conducted at 50 °C, which is expected as chlorination of terminal alkynes tends to be more challenging when compared to the other two transformations. This discovery of optimal conditions was carried over to test the tolerability scope of the reaction. Various functional group substituents, including electron-donating and -withdrawing groups on aryl alkynes, were tested, all resulting in excellent yields for the bromination (89 to 99%), iodination (88 to 99%), and chlorination (88 to 96%) (Scheme 13a–h). Through recyclability studies, the researchers reported their catalyst to demonstrate outstanding catalytic activity, stability, and the absence of Ag leaching. The catalyst is capable of being reused post-reduction over five times before any significant catalytic activity decrease is observed.

### 2.7. MOF Recyclable Catalysts

Metal-organic frameworks (MOFs) are crystalline solid-state materials composed of a porous framework built from strong bonds between inorganic (metal) units and organic linkers [21]. Recent advances in the use of MOFs for various applications from catalysis to storage, roots back to its simple ability to change its properties by controlling its shape, porosity, size, and functionality [22]. The tunability of these materials and their simple removal and reusability makes their role as recyclable heterogenous catalysts in organic synthesis very attractive. MOF systems have been applied and demonstrated to be useful as recyclable catalysts and catalytic support systems. The uniformity and adaptability of the pores and channels opens the door to use these materials for a myriad of transformations, including those related to the functionalization of alkynes, as demonstrated in the following work. 

**Scheme 14:**
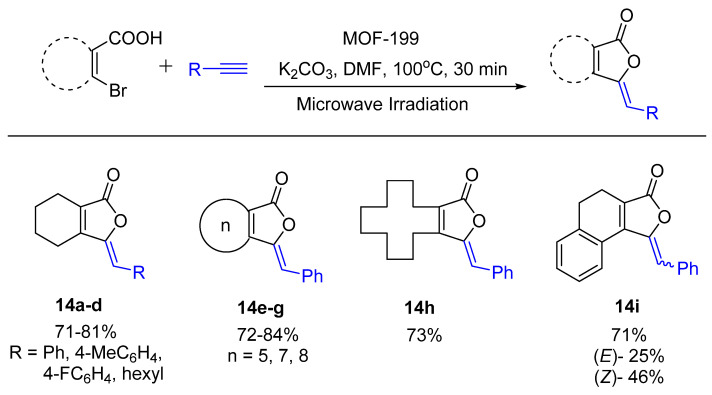
Reaction of β-bromo-α,β-unsaturated carboxylic acids with terminal alkynes to yield alkylidenefuranones through the use of MOF-199 as a recyclable catalyst (derived from [23]).

Ho et al. [23] reported the use and study of metal-organic framework MOF-199 as a recyclable catalyst for the efficient synthesis of alkylidenefuranones from the reaction of β-bromo-α,β-unsaturated carboxylic acids with terminal alkynes under microwave irradiation (Scheme 14).

Many furanone-containing compounds are known to show interesting biological activities [24] such as antibacterial, cytotoxic, antimicrobial, antibiotic, and antitumor activities. Prior work has shown the need for expensive transition metal catalysts for each transformation desired, posing potential drawbacks for the synthesis of these functionalized alkylidenefuranones [25]. The researchers conducted studies on the functional group tolerability for this transformation using this MOF catalyst with a wide substrate scope (Scheme 14a–i). Notably, both straight and branched alkyl substrates resulted in very good yields, especially when compared to those of previously published works giving lower yields. This increase in yield can be correlated to the increase in catalytic activity of MOF-199 for this transformation, indicating another advantage over previously used Pd on C/CuI/PPh_3_ and CuI/_L_-proline. According to the article, the MOF-199 catalyst has a simple recovery through classic filtration and simple solvent washing, allowing it to be used up to five times without catalytic activity degradation, contributing to its ability to act as a sustainable catalyst for the synthesis of alkylidenefuranones.

**Scheme 15:**
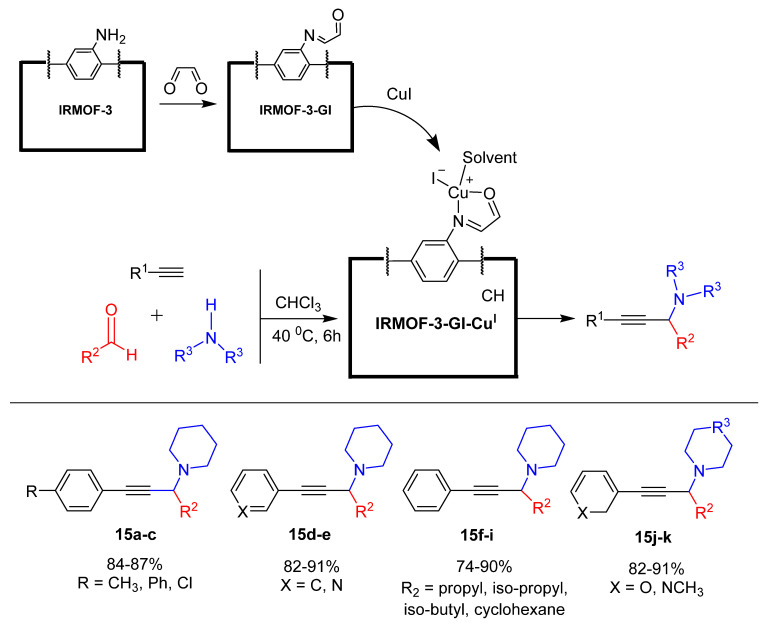
IRMOF-3-G1-Cu^I^ as a heterogeneous MOF-supported Cu(I) catalyst for three-component coupling reactions of alkynes, aldehydes, and amines (adapted from [26]).

The use of MOFs as recyclable catalysts for coupling reactions was also previously studied by Yang, Li, and Wang [26], who prepared IRMOF-3-G1-Cu^I^, a heterogenous MOF-supported Cu(I) catalyst, from the post-synthetic functionalization of IRMOF-3 with the ligand glyoxal and further treatment with CuI, in order to catalyze a three-component coupling reaction (Scheme 15).

As highlighted, this work studied the applications of this recyclable MOF catalyst in a three-component (A^3^) coupling reaction consisting of an alkyne, aldehyde, and amine. They used paraformaldehyde, phenylacetylene, and piperidine as model substrates in their investigations. To test the reaction scope, the researchers studied different arrangements of alkynes, aldehydes, and amines, varying one component substrate at a time and maintaining the other two model substrates constant. All substrate scope combinations resulted in excellent reported yields between 74 and 91% (Scheme 15a–k). The recoverability of the catalyst is reported to be a simple procedure: separation conducted through centrifuge after reaction completion, solvent washing, and air drying. Recyclability of the IRMOF-3-G1-Cu^I^ catalyst was also studied, and the researchers reported that it may be reused over four consecutive cycles.

### 2.8. POP Recyclable Catalysts

Porous organic polymers (POPs) are built from organic unit frameworks created and held together through strong covalent bonds [27]. The ability to manipulate and control the porosity, surface area, density, and functionality of POPs makes them best suited to work as efficient heterogenous catalysts, and therefore even better suited to be used as recyclable catalysts [28]. This broad array of tunable factors allows this category of recyclable catalysts to be used in a variety of transformations with practical and industrial applications. There are a variety of different types of POPs, such as polymers of intrinsic microporosity (PIMs) [29], hyper-crosslinked polymers (HCPs) [30], crystalline triazine frameworks (CTFs) [31], porous aromatic frameworks (PAFs) [32], conjugated microporous polymers (CMPs) [33], and covalent organic frameworks (COFs) [34].

**Scheme 16:**
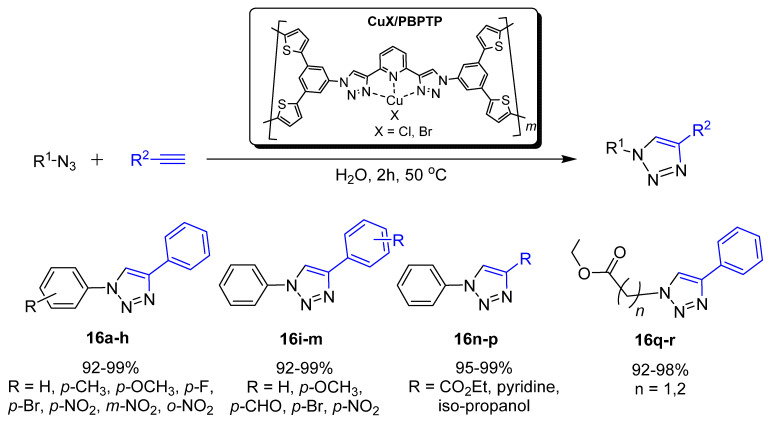
Azide-alkyne cycloaddition reactions catalyzed by porous polymer CuX/PBPTP (derived from [35]).

Most recently, in 2021, Cai et al. [35] reported the synthesis and catalytic utility of a porous organic polymer (POP) which was synthesized by thiophene-based oxidative coupling of [2,6-bis(1,2,3-triazol-4-yl)pyridine] (BTP) to produce 2,6-bis(1-(3,5-di(thiophen-2-yl)phenyl)-1*H*-1,2,3-triazol-4-yl)pyridine (PBPTP). This PBPTPB polymer was used as a ligand to form a coordinated complex with a copper salt, for the catalysis of azide-alkyne cycloaddition reactions (Scheme 16).

As shown, the coordination between the PBPTP motif and the copper salt, in this case either CuCl or CuBr, creates the center complex where the main reaction can occur in aqueous conditions. Preparation of the PBPTP polymer was carried out through the use of oxidative coupling in the presence of FeCl_3_. Following that, the CuX-PBPTP complex was created by combining the prepared PBPTP polymer with the desired CuX salt. Through optimization reactions, it was reported that the reaction best ran in water as the solvent, two hours of reaction time at 50 °C, and with the use of this CuX/PBPTP catalyst. Additional studies on the catalytic performance compared the reactions of other copper catalysts, such as CuBr_2_, CuCl, Cu(OAc)_2_, CuSO_4_, etc., to their complex system. Based on the resulting yields (38 to 65%) they concluded their heterogenous catalyst to be more efficient. They also tested the use of PBPTP alone without the presence of a coordinated complex, and found that the yield was 2%, indicating the need for their CuX-PBPTP complex and the crucial catalytic role it takes in the reaction. 

Based on the substrate scope studied, this reaction supports several functionalities of azide and alkyne substrates. Phenylacetylene was used as a constant substrate to test various benzyl azides with electron-donating and -withdrawing groups attached; these reactions resulted in excellent yields between 92 and 99% (Scheme 16a–h), with the latter demonstrating greater reactivity. The *o-*, *m*-, and *p*-positions on the benzene ring were also tested and, based on the yields steric hindrance, may have a slight effect on the yield. An aliphatic azide group was also tested with positive results of 92 to 98% yield (Scheme 16q–r). Likewise, benzyl azide was used as a constant substrate to test various terminal alkyne substrates, including aromatic substrates, with 92 to 99% yields (Scheme 16i–m), and aliphatic substrates, with 95 to 99% yields (Scheme 16n–p). The researchers suggest that bulky substrates may result in lower yields due to the fact that the catalytic site is within the pores of the CuX-PBPTP, where the reaction is indicated to take place. The catalyst was effortlessly removed after the reaction completion through filtration, allowing it to be recycled. Based on the ICP-AES analysis conducted, there was no observed Cu leaching of the catalyst, no substantial decrease in catalytic activity, and it can be reused more than six times. For these reasons, this category of complex catalyst systems will open the doors to many future systems, leading as a model for others to be used for other organic transformations, and may pave the way to a greener synthetic future.

### 2.9. Bio-Supported Catalyst Systems

The incorporation of biomaterials in catalysis is a field of materials chemistry that has long been evolving. With this review’s focus on the use of recyclable catalysts for the functionalization of alkynes, it would be inevitable to mention the use of biomaterials for the development of new recyclable catalysts.

**Scheme 17:**
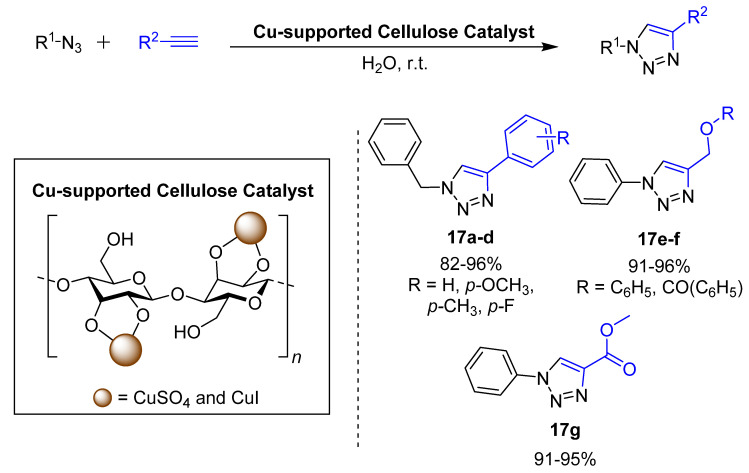
Cu-supported cellulose recyclable catalyst for azide-alkyne cycloaddition (CuAAC) click chemistry reactions (derived from [36]).

In 2018, Bahsis et al. [36] reported on the use of a natural macromolecule, cellulose, that could be immobilized with copper ions to create cellulose-supported copper recyclable catalysts, which would facilitate Cu-catalyzed azide-alkyne cycloaddition (CuAAC) click chemistry reactions in water (Scheme 17).

The bio-supported Cu-catalyst was synthesized by taking extracted natural cellulose and adding it to a solution of desired copper ions. In this study, the researchers synthesized and tested both cellulose-supported CuSO_4_ and CuI catalysts using the same technique but changing the desired copper supply. To test the catalytic scope of both cellulose-supported copper catalysts, several substrates were tested with each catalyst, both of which resulted in good to excellent yields. All substrates were tested with benzyl azide. Aryl substituents consisting of phenylacetylene and either electron-withdrawing or -donating groups in the *para* position were well-tolerated with yields between 82 and 96% (Scheme 17a–d). Aliphatic groups with benzene-containing (-R) groups yielded between 91 and 96% (Scheme 17e–f). Finally, terminal alkynes attached to ester groups resulted in 91 to 95% yields (Scheme 17g) when tried with both catalysts. Recovery and recyclability studies were conducted on the cellulose-supported copper heterogeneous catalysts using the model reaction of phenylacetylene and benzyl azide. Simple recovery of the catalyst after the termination of the reaction was carried out through simple filtration using diethyl ether to wash and dried afterwards, allowing it to be ready for the following use. The studies indicated that this catalyst could be recycled in five runs with an extremely low effect on yields. The researchers also used atomic absorption spectroscopy to test the heterogeneous properties and the presence of copper leaching for the catalyst. To their delight, there were very minimal traces of copper detected to be leaching, allowing for this catalyst to be acceptable to implement in pharmaceutical practices. 

### 2.10. Solvent and Metal-Free Recyclable Catalysts

It is important to note that the use of simple chemical reagents can be manipulated to serve as recyclable catalysts to carry out “greener” alternative reaction transformations. The ideals of green chemistry promote the development of reaction methodologies with minimal reagents, the absence of toxic metals, high selectivity, minimal waste, mild conditions, and atom economy.

Yamada et al. [37] developed a mild and efficient approach for the deuteration of terminal alkynes through the use of heterogeneous basic polystyrene resin (WA30) under mild conditions in the presence of D_2_O. Based on their findings, a wide range of functionalities such as nitro, propargyl ester, sulfide, benzyl ether, and others were very well-tolerated under such conditions. Recyclability studies of this resin material found that it could be recovered and reused frequently without any deprivation in catalytic activity. This economic and eco-friendly deuteration method can be applied in both laboratory and industrial settings due to its recyclability and green chemical approach. It can also serve as a foundation for the discovery of more alternative deuteration and functionalization approaches using environmentally favorable methods.

**Scheme 18:**
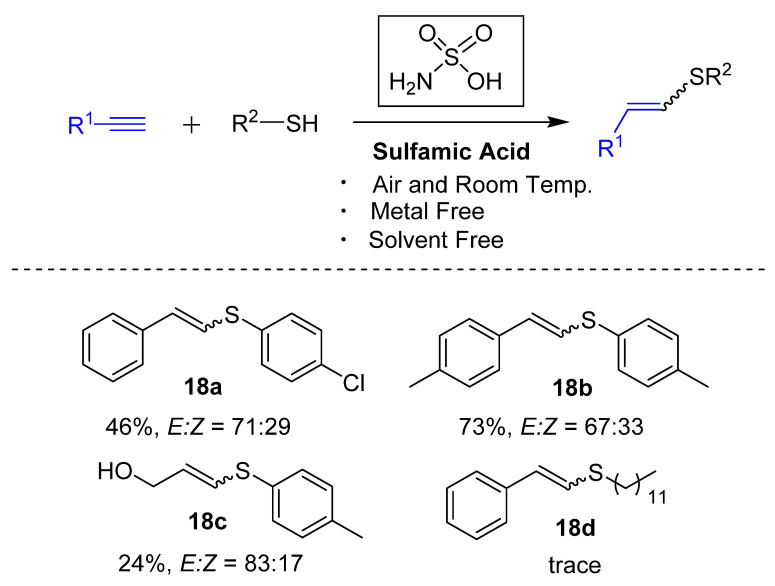
Metal- and solvent-free synthesis of thioesters via regioselective alkyne hydrothiolation using sulfamic acid (adapted from [38]).

In 2017, Rosa et al. [38] reported on their use of sulfamic acid as an efficient recyclable catalyst to promote the formation of thioesters through regioselective hydrothiolation of alkynes, utilizing ideal conditions under air and at room temperature, in a metal- and solvent-free method (Scheme 18).

In this study, they utilized sulfamic acid, which is a simple, environmentally friendly, affordable, and readily available chemical, which showed great catalytic activity for this transformation. To study the limitations of their metal- and solvent-free reaction, they tested a wide range of terminal alkynes and thiols to synthesize different vinyl thioether products. Based on the results from their substrate scope study, the reaction had a high stereoselectivity, even in the case with less reactive aliphatic thiols (Scheme 18c). The aromatic substituent’s electronic effects for the terminal alkynes were also studied to test for reaction tolerability and were shown to result in decent yields (Scheme 18a,b). This was not the case with aliphatic alkynes, where little to no reaction was observed (Scheme 18d). This catalyst can be easily recovered via simple filtration after reaction completion and, after several studies, was found to be reusable up to four times before noticeable catalytic activity loss results in a decreased reaction yield. This technique of using a simple reagent to perform a clean and sustainable synthetic reaction, without the need of complicated and harsh reaction conditions, is a motivation for the discovery of future alternative environmentally friendly methods for other necessary organic reactions.

## 3. Conclusions

As seen in the current work presented within this review, the use of recyclable catalysts for alkyne functionalization is currently evolving and growing. Due to the interest of developing and discovering recyclable systems, the broad spectrum of applications for this type of catalysis is ever increasing. Herein, we have highlighted several categories of recyclable catalysts, such as polysiloxane-encapsulated metal nanoparticle-based catalysts, silica–copper-supported nanocatalysts, graphitic carbon-supported nanocatalysts, metal organic framework (MOF) catalysts, porous organic framework (POP) catalysts, biomaterial-supported catalysts, and metal/solvent free recyclable catalysts. Along with the variety of catalytic systems, several alkyne functionalization reactions have been elucidated to demonstrate the success and efficiency of recyclable catalysts in carrying out these reactions in comparison to traditional techniques. Each reported catalyst has resulted in transformations of high yields and selectivity. They have each shown their ability to be recovered and recycled from the reaction, up to or, in several cases, more than four times. We hope that the works highlighted in this review will serve as a motivation to continue the advancements of these catalysts. With the focus on green chemistry, competitive efficiency, and the advantage of simple recovery and reusability, these catalysts pose a large benefit and advantage for future applications in industry and academic research.
